# Economic and epidemiological impact of early antiretroviral therapy initiation in India

**DOI:** 10.7448/IAS.18.1.20217

**Published:** 2015-10-01

**Authors:** Manoj V Maddali, David W Dowdy, Amita Gupta, Maunank Shah

**Affiliations:** 1 Division of Infectious Diseases, Department of Medicine, School of Medicine, Johns Hopkins University, Baltimore, MD, USA; 2 Department of Epidemiology, Bloomberg School of Public Health, Johns Hopkins University, Baltimore, MD, USA; 3 Department of International Health, Bloomberg School of Public Health, Johns Hopkins University, Baltimore, MD, USA

**Keywords:** HIV, India, cost-effectiveness, antiretroviral therapy, continuum of care

## Abstract

**Introduction:**

Recent WHO guidance advocates for early antiretroviral therapy (ART) initiation at higher CD4 counts to improve survival and reduce HIV transmission. We sought to quantify how the cost-effectiveness and epidemiological impact of early ART strategies in India are affected by attrition throughout the HIV care continuum.

**Methods:**

We constructed a dynamic compartmental model replicating HIV transmission, disease progression and health system engagement among Indian adults. Our model of the Indian HIV epidemic compared implementation of early ART initiation (i.e. initiation above CD4 ≥350 cells/mm^3^) with delayed initiation at CD4 ≤350 cells/mm^3^; primary outcomes were incident cases, deaths, quality-adjusted-life-years (QALYs) and costs over 20 years. We assessed how costs and effects of early ART initiation were impacted by suboptimal engagement at each stage in the HIV care continuum.

**Results:**

Assuming “idealistic” engagement in HIV care, early ART initiation is highly cost-effective ($442/QALY-gained) compared to delayed initiation at CD4 ≤350 cells/mm^3^ and could reduce new HIV infections to <15,000 per year within 20 years. However, when accounting for realistic gaps in care, early ART initiation loses nearly half of potential epidemiological benefits and is less cost-effective ($530/QALY-gained). We project 1,285,000 new HIV infections and 973,000 AIDS-related deaths with deferred ART initiation with current levels of care-engagement in India. Early ART initiation in this continuum resulted in 1,050,000 new HIV infections and 883,000 AIDS-related deaths, or 18% and 9% reductions (respectively), compared to current guidelines. Strengthening HIV screening increases benefits of earlier treatment modestly (1,001,000 new infections; 22% reduction), while improving retention in care has a larger modulatory impact (676,000 new infections; 47% reduction).

**Conclusions:**

Early ART initiation is highly cost-effective in India but only has modest epidemiological benefits at current levels of care-engagement. Improved retention in care is needed to realize the full potential of earlier treatment.

## Introduction

In 2013, there were 2.1 million new HIV infections worldwide, a 38% decrease in yearly incidence from 2001. This progress stems from immense global investment in HIV prevention efforts and subsequently increased availability of antiretroviral therapy (ART) for treatment of HIV infection [1]. Early ART initiation at higher CD4 cell counts has been shown to prolong immunologic function and reduce HIV transmission among people living with HIV (PLWH), as individuals with sustained viral suppression are unlikely to transmit HIV to sexual partners [1–3]. Prior models have suggested that “test-and-treat” policies implementing universal HIV testing with immediate ART initiation may drastically reduce HIV prevalence [4]. In light of this potential patient and public health benefit, recent World Health Organization (WHO) treatment guidelines advocate for early ART initiation at higher CD4 counts (≤500 cells/mm^3^) [5].

Attrition of the HIV care continuum, however, can limit the impact of early ART initiation. Despite India's recent successes in slowing its national HIV epidemic, national HIV screening rates remain low at 3.2% per year [6]. Consequently, many Indians are unaware of their HIV serostatus and nearly 20% of PLWH present to care with late diagnosis (CD4 ≤50 cells/mm^3^) [7]. Even after linkage to care and ART initiation, poor patient adherence, treatment fatigue and various sociocultural barriers can lead to suboptimal retention in care [8–11]. Ultimately, less than one third of Indian adults diagnosed with HIV currently achieve viral suppression [12].

Long-term engagement in HIV care is especially critical to realize the full impact of new ART treatment guidelines, as earlier treatment may increase opportunities for intermittent episodes of disruption in care and development of ART resistance [13]. Prior models have suggested that early ART initiation in India is cost-effective, but have not fully considered the degree to which suboptimal engagement in the HIV care continuum could attenuate economic and health benefits of earlier ART initiation [14,15]. We thus sought to evaluate the impact of early ART initiation in India within the context of the full HIV care continuum. We utilized a dynamic transmission model of the Indian HIV epidemic to provide quantitative estimates of how the economic and epidemiological impact of early ART initiation (compared to ART initiation at CD4 ≤350 cells/mm^3^) is modified by attrition throughout the HIV continuum of care.

## Methods

### Overview

Our primary outcomes were HIV prevalence and incidence, AIDS-related deaths, quality-adjusted-life-years (QALYs) and HIV-related healthcare costs for Indian adults over a 20-year time horizon. We evaluated how the impact of an early ART intervention (compared to current Indian HIV care practices initiating ART at CD4 ≤350 cells/mm^3^) was modified by the HIV continuum of care [16]. We defined early ART initiation as initiation of ART at CD4 ≥350 cells/mm^3^ at a rate defined in Table 1. Specifically, we first assessed the incremental costs and effects of early ART initiation compared to current practice of ART initiation at CD4 ≤350 cells/mm^3^ in the context of an “idealized” care continuum (under conditions of high rates of screening, linkage, adherence, treatment modification and retention in care; Table 1). Next, we examined the degree to which the epidemiological and economic impact of early ART initiation (compared to current practice) was modified when considering current realistic gaps in HIV care (Table 1). We also quantitatively explored the degree to which various stages of the care continuum can modulate the epidemiological impact of an early ART initiation policy.

**Table 1:** Key model parameters

Variables	Value	Sensitivity analysis	References
**HIV disease dynamics without antiretroviral therapy (ART)**			
Duration of acute HIV infection	2.9 months	1–4 months	[17,18]
Duration of early HIV infection: CD4 >350 cells/mm^3^	6.5 years	4–10 years	[19–21]
Duration of late HIV infection: CD4 200–350 cells/mm^3^	2.5 years	1–5 years	[19,20]
Duration of AIDS CD4 ≤200 cells/mm^3^ (until death)	1.5 years	1–5 years	[17,18,21–24]
Excess HIV mortality not on ART CD4 >200 cells/mm^3^	0.14% per year	0.1–1% per year	[25–27]
**HIV disease dynamics with ART**			
Reduction in rate of transmission	93%	80–99.5%	[3,28–30]
Time to viral suppression on ART	4 months	2–12 months	[23]
Reduction in rate of AIDS death on ART (CD4 ≤200 cells/mm^3^)	90%	50–95%	[24,31]
**Transmission dynamics^a^**			
Annual partnerships per year	0.45–6.2	0.25–8	[32], calculated
Transmission per partnership (male to female)	6%	4.5–7.5%	[28], calculated
Transmission per partnership (female to male)	4%	2.5–5.5%	[28], calculated
Transmission per partnership (MSM)	7%	5.5–8.5%	[28], calculated
Transmission per partnership (FSW)	1.875%	1–3%	[28], calculated
Transmission probability per shared needle (PWID)	0.23%	0.1–0.75%	[33,34]
Relative risk increase in transmission probability during acute HIV	12	2–4	[17,18]
**HIV care continuum dynamics with current, “realistic” gaps in care^b^**			
Percentage of HIV testing in past 12 months	3.2–31.8%	1–60%	[6]
Percentage of newly diagnosed HIV patients linked to care	55–80%	25–100%	[12]
Rate of disengagement from care annually	0.15–0.195	0.075–0.39	[12,35,36], assumption
Rate of reengagement in care annually	0.33	0.165–0.66	[12,35,36], assumption
Percentage of PLWH who develop resistance to first-line ART after dissengagement	25%	10–50%	[37], assumption
Annual failure rate of ART	0.07–0.1 yearly	0.02–0.3 yearly	[35,38]
Rate of ART failure identification and treatment modification	0.5–0.8 yearly	0.05–1.5 yearly	[35,38]
Rate of annual early ART initiation with CD4 ≥350 cells/mm^3^	2	0.25–4	
**Costs ($, USD 2014)^c^**			
Voluntary Counseling and Testing	$4.74	$1–$10	[39]
HIV viral load	$48.65	$20–$100	[40]
CD4 test	$6.63	$3–$15	[40]
Outpatient clinic visit	$3.17	$1–$15	[41]
Annual first-line ART	$133.40	$50–$300	[16,42], calculated
Annual second-line ART	$328.80	$100–$700	[16,42], calculated
**Utility weights**			
Uninfected	1	–	
Acute HIV	0.84	0.8–0.9	[43,44]
HIV unsuppressed CD4 >350 cells/mm^3^	0.94	0.9–0.99	[43,44]
HIV unsuppressed CD4 200–350 cells/mm^3^	0.84	0.8–0.99	[43,44]
HIV/AIDS unsuppressed CD4 ≤200 cells/mm^3^	0.78	0.5–0.9	[43,44]
Reduction in disability with viral suppression	75%	0–90%	[Assumption]
Usage of ART	0.98	0.94–1	[43,44]

a The numbers of annual partnerships and probability of transmission per partnership were calibrated to reflect published literature on HIV prevalence and incidence in India and were varied by gender and risk group. The probability of transmission was also influenced by condom usage, male circumcision, stage of HIV disease and awareness of HIV serostatus (see Supplementary file).

b Annual rates of HIV screening, linkage to care and disengagement from care were varied by gender and risk group. HIV screening was varied among risk groups based on national estimates; we also incorporated symptomatic screening. Linkage to care was defined as an HIV clinic visit within three months of diagnosis. Reengagement is defined as a return to care among PLWH aware of serostatus but not in care. In the “idealized” care continuum scenario, we assumed annual screening of high-risk groups with 95% linkage to care within three months, lower rates of ART failure due to improved adherence (0.03–0.05 yearly), identification of ART failure and subsequent treatment modification within one year, and optimal retention in care (annual disengagement rate of 2.5% per year and reengagement within one year of disengagement).

c We included additional annual healthcare utilization costs for PLWH not in care or on ART (e.g. hospitalizations; see Supplementary file). First-line therapy ART was assumed to include tenofovir (TDF), lamivudine (3TC), and efavirenz (EFV). Second-line ART regimen was assumed to include zidovudine (AZT), lamivudine (3TC) and lopinavir/ritonavir (LPV/r).

### Model structure

**Figure 1:**
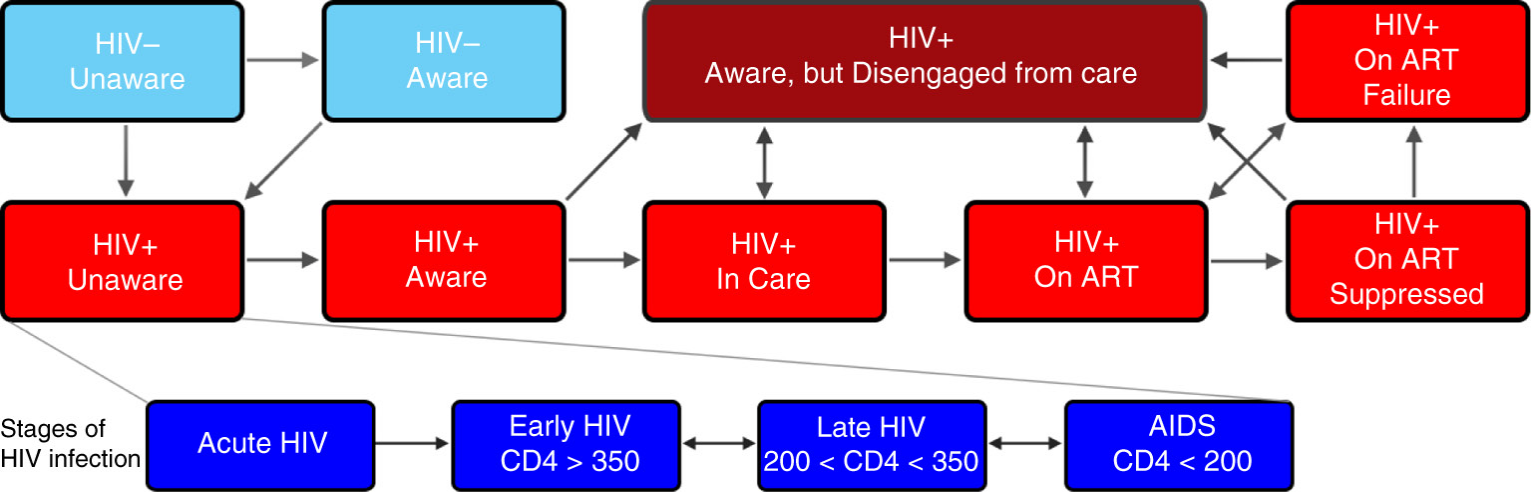
Model schematic of HIV transmission, disease progression and engagement in HIV care continuum. The Indian population is divided into compartments stratified by HIV serostatus, stage of HIV infection, and engagement with HIV care continuum. Each compartment is further stratified by gender and risk group (heterosexual, MSM, PWID and high-risk individuals [e.g. FSW]). The model incorporates HIV transmission through sexual intercourse and injection drug use. Arrows represent rates of flow between compartments; values are shown in Table 1. +, HIV-infected individuals; −, HIV-uninfected individuals; ART, HIV-infected individuals on first- or second-line antiretroviral therapy. At any point in the care continuum, PLWH can progress through stages of HIV from acute HIV to AIDS if not on ART (shown in inset). Individuals experience immunological recovery if on ART and virologically suppressed.

We constructed a dynamic compartmental model of the Indian HIV epidemic that incorporates transmission, disease progression and health system engagement (Figure 1). In our model, India's adult population (15–64 years) is divided by sex (male or female), HIV risk-profile (heterosexuals, men who have sex with men (MSM), people who inject drugs (PWID), female sex workers (FSW), and high-risk males) and HIV infection status. HIV transmission in our model occurs through sexual contact (heterosexual or male homosexual) and needle sharing among PWID. Risk of HIV transmission was influenced by frequency of sexual interactions and needle sharing within and across risk groups, stage of HIV infection (e.g. higher transmission potential during acute HIV) and ART usage (Table 1).

Upon infection with HIV, PLWH progressed through a series of compartments based on disease progression (stratified by CD4 count) and engagement with the care continuum (e.g. unaware of HIV status, diagnosed but not in care, in care but not on ART, on ART but not virologically suppressed, on ART but experiencing virological failure, virologically suppressed, ART regimen; Figure 1). For those on ART, we considered both first- and second-line regimens. We estimated rates of virological failure, detection and treatment modification to second-line regimens from published data [35,38]. We calibrated the model to reflect estimates of HIV prevalence and incidence in India [45]. Rates of flow between model compartments were represented by a system of ordinary differential equations (see Supplementary file).

### Economic impact and cost-effectiveness

We calculated healthcare costs and QALYs utilizing a unit-costing approach, estimating the number of person-years spent in each HIV-related model compartment, including costs associated with transitions between model compartments (e.g. becoming aware of serostatus after HIV testing). Costs are reported in 2014 US dollars (USD); in primary analysis future costs and QALYs were discounted 3% [46]. Costs from prior years were converted into Indian currency (INR) using year-specific exchange rates and were inflated according to published estimates of Indian inflation rates [21]. Unit costs, utility weights and other key model parameters are shown in Table 1.

We defined cost-effectiveness based on WHO Commission on Macroeconomics and Health willingness-to-pay threshold recommendations, which define Incremental Cost-Effectiveness Ratios (ICER) <3× the annual GDP per capita as “cost-effective” and ICERs <1× the annual GDP per capita as “very cost-effective” (2014 Indian GDP per capita is $1584) [47,48].

### Model calibration and sensitivity analysis

We calibrated HIV transmission probabilities within risk groups and rates of engagement in care and treatment initiation to generate model outputs that best reflected reported epidemiological data on HIV prevalence, incident cases, care continuum engagement and PLWH on ART in India between 2007 and 2011 (the last year for which there was complete data; see Supplementary file) [7,12,45].

We conducted one-way sensitivity analyses on all parameter values (Table 1) and report on the parameters that most influenced final estimates of cost-effectiveness of early ART initiation in a “realistic” care continuum. We also conducted probabilistic uncertainty analysis for both “idealized” and “realistic” care continuum scenarios by simultaneously varying all parameter values by Latin Hypercube sampling over specified ranges to generate 95% uncertainty ranges (URs), reported as the 2.5th and 97.5th percentiles.

## Results

Assuming optimal levels of engagement throughout the care continuum (i.e. from diagnosis to virological suppression), we project 831,000 new HIV infections (95% UR 561,000–1,447,000) and 482,000 AIDS-related deaths (95% UR 427,000–821,000) would occur in India over 20 years if current ART initiation practices (CD4 ≤350 cells/mm^3^) were maintained (Table 2). Early ART initiation in this idealized care continuum would result in 517,000 new infections (38% reduction; 95% UR 330,000–896,000) and 411,000 AIDS-related deaths (15% reduction; 95% UR 341,000–652,000) over two decades, at a cost-effectiveness of $442/QALY-gained (95% UR $181–$693) and incremental healthcare expenditures of $329 million (95% UR $239–$784 million; $388 million undiscounted). With optimal care-engagement, early ART initiation could reduce annual new HIV infections to <15,000 per year within 20 years and would be considered highly cost-effective.

**Table 2:** Key model outputs assessing the modulatory effect of the HIV continuum of care on the impact of early ART initiation (compared to current practices of ART initiation at CD4 ≤350 cells/mm^3^)

	Idealistic continuum of care^a^		Realistic continuum of care^a^	
		
	Delayed ART initiation (95% UR)^b^	Early ART initiation (95% UR)^b^	Delayed ART initiation (95% UR)^b^	Early ART initiation (95% UR)
New HIV infections	831,000 (reference) (561,000–1,447,000)	517,000 (38% reduction) (330,000–896,000)	1,285,000 (Reference) (876,000–2,114,000)	1,050,000 (18% reduction) (706,000–1,729,000)
AIDS-related deaths	482,000 (reference) (427,000–821,000)	411,000 (15% reduction) (341,000–652,000)	973,000 (reference) (679,000–1,412,000)	883,000 (9% reduction) (610,000–1,300,000)
Incremental costs ($USD)	Reference	$329 million ($239–$784 million)	Reference	$400 million ($245–$745 million)
ICER ($USD/QALY-gained)	Reference	$442/QALY-gained ($181–$693)	Reference	$530 QALY-gained ($301–$1010)

UR, uncertainty range; ART, anitretroviral therapy; USD, US dollars; ICER, incremental cost-effectiveness ratio; QALY, quality-adjusted-life-years.

a “Realistic” continuum of care incorporates current rates of attrition in HIV screening, linkage, adherence, and retention in care. “Idealistic” continuum of care assumes optimized HIV care delivery, with annual HIV screening for high-risk populations, improved linkage to care, lower rates of ART resistance due to improved adherence, and faster detection of failure, and optimal retention in care.

b The 2.5th and 97.5th percentiles for uncertainty ranges (URs) for all key model outputs are shown in parentheses. Delayed ART is defined as continuation of current practices of ART initiation (CD4 ≤350 cells/mm^3^), and early ART initiation is defined as initiating ART at higher CD4 counts (CD4 ≤500 cells/mm^3^).

Accounting for current attrition in HIV care resulted in poorer outcomes of the Indian HIV epidemic. With realistic gaps in care (e.g. poor retention leading to ART resistance), projections of 20-year outcomes with delayed ART initiation (CD4 ≤350 cells/mm^3^) rose to 1,285,000 new HIV infections (95% UR 876,000–2,114,000) and 973,000 AIDS-related deaths (95% UR 679,000–1,412,000). If current levels of engagement in care persist, we project that the Indian healthcare system would incur costs of $9.6 billion (with 3% discounting) for HIV-related expenses (95% UR $6.4–$17.3 billion; $13.0 billion without discounting) with ART delayed to CD4 ≤350 cells/mm^3^. In this setting, we estimated that 31% of PLWH would require post-first-line regimens in 20 years.

Incorporating these current real-world gaps in HIV care-engagement halved the epidemiological impact of early ART initiation. If implementing early ART initiation within the current care continuum, we estimated 1,050,000 new HIV infections (95% 706,000–1,729,000) and 883,000 AIDS-related deaths (610,000–1,300,000) over two decades, or 18 and 9% reductions (respectively) compared to ART initiation at CD4 ≤350 cells/mm^3^. Earlier treatment in a realistic care continuum resulted in 20-year incremental costs of $400 million (95% UR $245–$745 million; $517 million undiscounted). Despite diminished epidemiological impact, early ART initiation remains cost-effective compared to delayed ART initiation even when accounting for the current HIV care continuum ($530 per QALY-gained; 95% UR $301–$1010) though at a less favourable cost-effectiveness ratio (20% increase compared to cost-effectiveness estimates under the idealized care continuum scenario; Table 2).

With earlier ART initiation, individuals are projected to have longer time periods on ART with increased opportunities for virological failure and disengagement from care; we project that in 20 years, 38% of PLWH would require post-first-line regimens with earlier ART initiation. The potential impact of early treatment was further compromised by attrition throughout all stages of the care continuum. Regardless of an early ART initiation policy, we project that over half of PLWH (53%) on average would present to care with CD4 ≤350 cells/mm^3^, and only 59% of PLWH who linked to care (and 44% of all PLWH) achieved virological suppression over 20 years.

**Figure 2:**
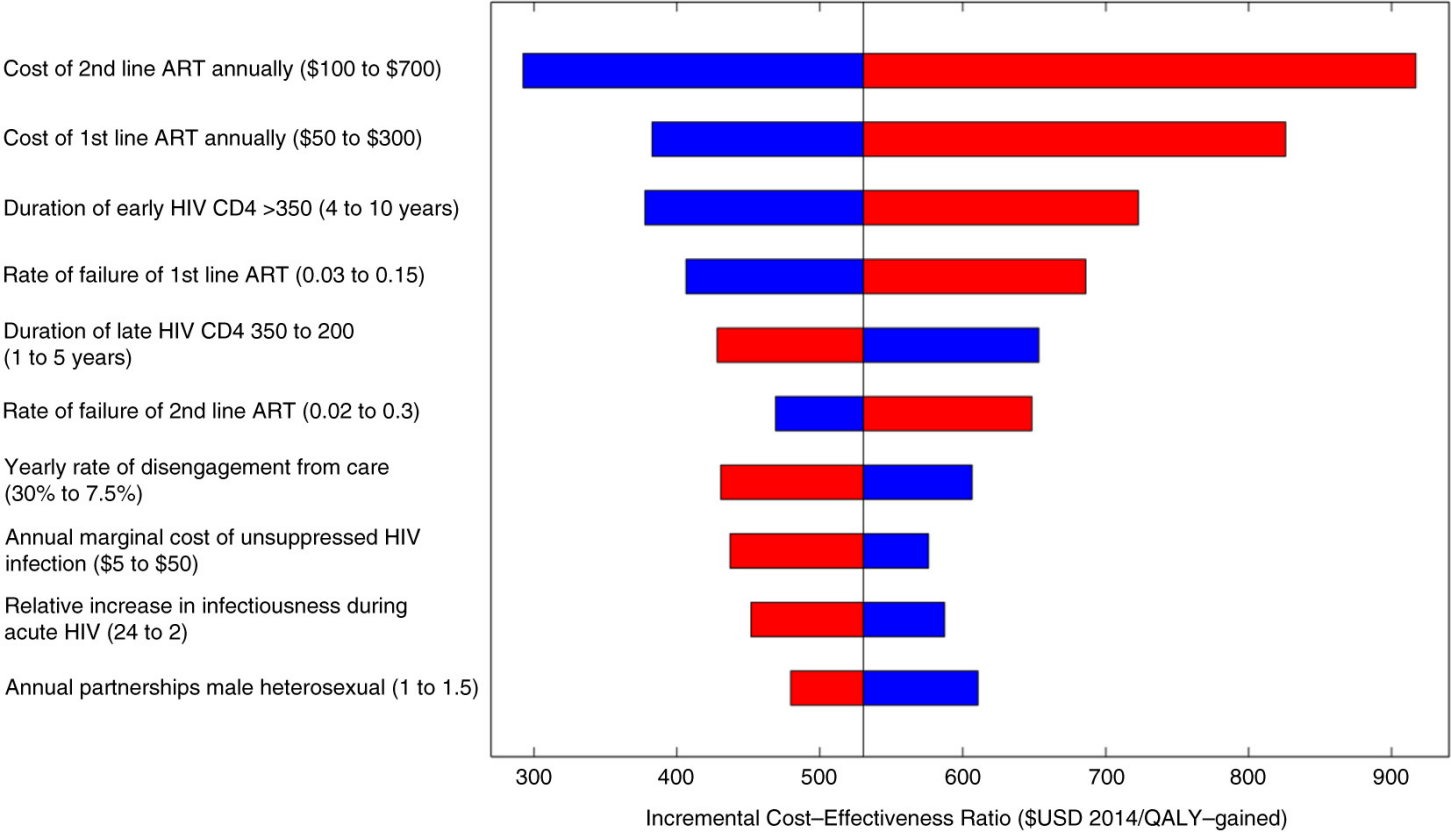
Sensitivity analysis for incremental cost-effectiveness ratio comparing early ART initiation with current practices of ART initiation (CD4 ≤350 cells/mm^3^) within the context of a “realistic” continuum of care. Solid vertical line represents base ICER ($530 per QALY-gained). Blue bars indicate low values of parameter range; red bars indicate high values of parameter range.

In one-way sensitivity analysis, ART costs and failure rates were key drivers of the epidemiological impact and cost-effectiveness of early treatment within the current care continuum (Figure 2). However, early ART initiation was very cost-effective compared to initiation at CD4 ≤350 cells/mm^3^ in most scenarios including at higher estimates of HIV healthcare-related costs. For example, even at the highest estimated annual costs for both first and second-line ART, early ART initiation remained very cost-effective at $1212 per QALY-gained. Additionally, earlier treatment remained very cost-effective even at higher estimates of ART failure rates (ICER $903 per QALY-gained).

**Figure 3:**
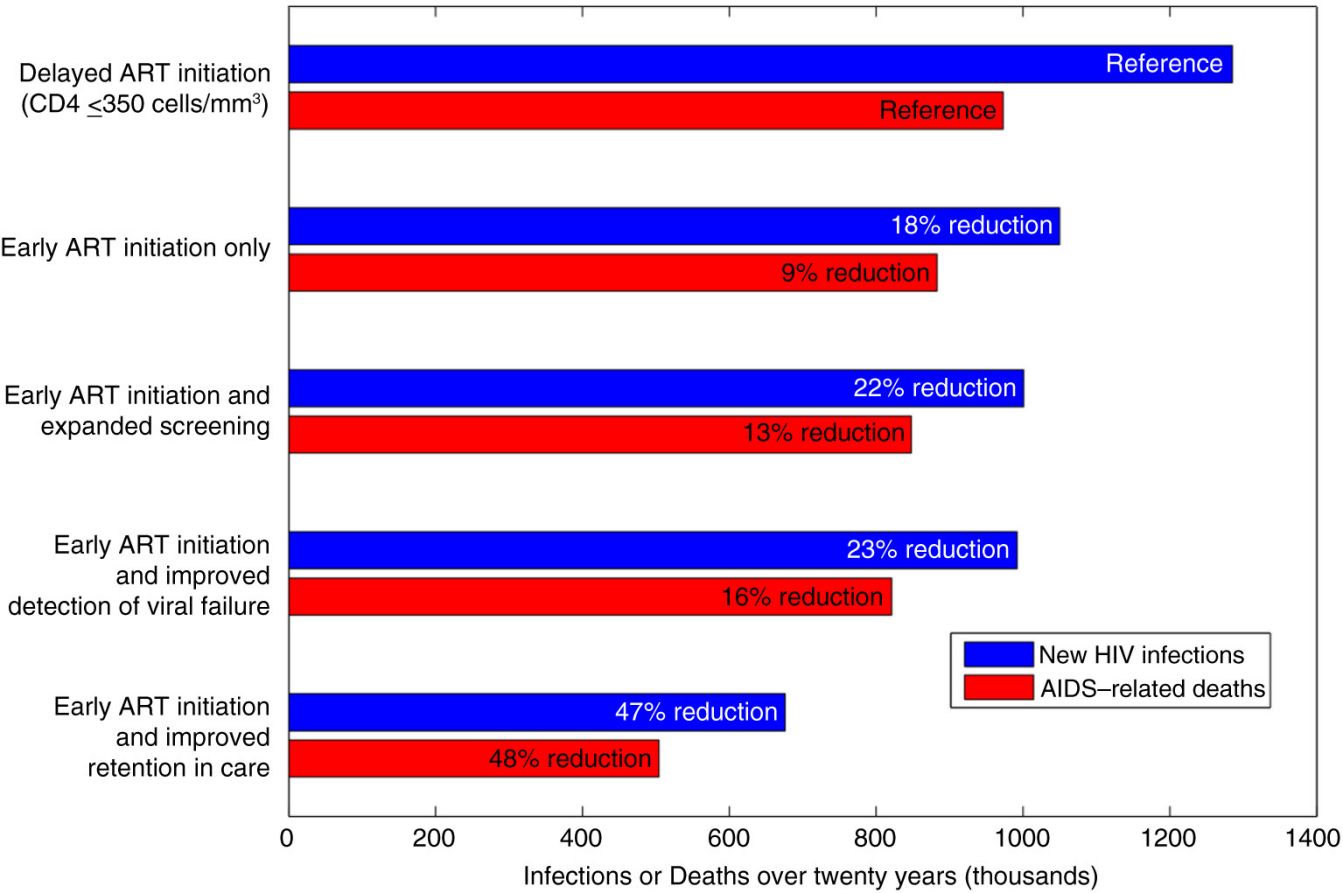
Epidemiological impact of delayed ART initiation (CD4 ≤350 cells/mm^3^), early ART initiation and early ART initiation in combination with various interventions in the HIV care continuum. “Delayed ART initiation” represents current practice and incorporates the current HIV care continuum with suboptimal screening, linkage and retention in care. “Early ART initiation” represents increased rates of ART initiation at CD4 >350 cells/mm^3^, but assumes continuation of current levels of care-engagement. “Expanded screening” involves annual screening of high-risk groups, with 95% linkage to care. “Improved detection of virological failure” involves detecting ART failure and modifying treatment promptly (within six months of virological failure). “Improved retention in care” is defined as optimal retention of PLWH in care (annual disengagement rate of 2.5% and reengagement within one year of disengagement).

We found that the degree to which each stage of the care continuum modifies the epidemiological impact of early HIV treatment in India can vary greatly (Figure 3). For example, if early treatment was combined with rapid identification of ART failure (with prompt changes to alternative effective regimens, i.e. second-line therapy), our model projects 992,000 new infections (23% reduction) and 821,000 AIDS-related deaths (16% reduction) over 20 years, despite other gaps in care (e.g. poor linkage and retention).

Implementing early ART initiation with expanded screening and linkage for high-risk groups (i.e. test-and-treat strategies) also offered relatively modest benefits at current rates of retention in care, with 1,001,000 new infections (22% reduction) and 848,000 AIDS-related deaths (13% reduction) projected over 20 years. Similar to prior models, we determined that annual targeted screening with early ART initiation is highly cost-effective in India ($1242/QALY-gained), even when accounting for suboptimal care-retention [14]. However, our model suggests that expanded screening among the general population (in addition to earlier HIV treatment and targeted screening for high-risk groups) is unlikely to be cost-effective in the current Indian setting ($5368/QALY-gained).

Overall, to achieve significant population-level impact, we found that early ART initiation would need to be combined with improved retention in care after linkage. If long-term retention of PLWH in care were achieved (i.e. reductions in yearly rates of disengagement and improved reengagement), we project only 676,000 new HIV infections (47% reduction) and 504,000 AIDS-related deaths (48% reduction) would occur over 20 years with policies and provisions for earlier treatment.

## Discussion

Recent WHO recommendations call for earlier ART initiation at higher CD4 thresholds compared to current Indian guidance delaying ART until CD4 ≤350 cells/mm^3^ [5,16]. When accounting for attrition throughout the continuum of HIV care, our model suggests that early ART initiation has attenuated benefits in reducing India's HIV epidemic, compared to estimates derived when assuming “optimal” engagement in HIV care (e.g. high rates of screening, linkage, adherence and retention in care). In particular, we found that assumptions of idealistic HIV care-engagement would lead to twofold overestimation of the epidemiological impact of early ART initiation. However, despite care attrition and diminished epidemiological impact, earlier treatment remains highly cost-effective and is within the financial scope of current Indian HIV expenditures.

Despite recent scale-up of ART availability, high proportions of PLWH remain unaware of their serostatus or have delayed presentations to care with advanced immunosuppression [7]. Moreover, current data suggest that many PLWH become disengaged from long-term care, resulting in on-going transmission and excess mortality from HIV [10–12]. Consequently, even if provisions are made for early ART initiation, many potential benefits will remain unfulfilled. Our results suggest that more than half of PLWH would present to care at CD4 counts below the threshold for current treatment initiation (CD4 ≤350 cells/mm^3^) and would therefore fail to derive the benefits of an early ART initiation policy.

Our model is unique in quantifying the degree to which suboptimal engagement in care modulates the impact of early ART initiation. In contrast to the modest effects of earlier treatment with attrition in HIV care, early ART initiation in the context of an optimized care continuum could reduce annual HIV transmissions in India by nearly 90%, from 120,000 yearly infections currently to <15,000 infections per year within two decades [1]. Efforts are therefore urgently needed to identify evidence-based strategies to strengthen HIV healthcare systems. Our results suggest that ensuring retention in care is crucial to achieving “treatment as prevention” through long-term viral suppression and reducing emergence of drug resistance. In contrast, while expanding HIV screening among high-risk groups is important and effective, the overall epidemiological benefit of such strategies hinges on the ability to retain patients in care.

Improving retention in care will require a concerted effort from care-providers, HIV programmes and policy-makers. Decentralization of ART distribution networks has been shown to decrease disengagement rates in rural settings [49]. India's rapid scale-up of ART has increasingly shifted the burden of ART distribution from large care centres to local dispensaries and clinics, with promising results [7,50]. Robust patient management and tracking systems will need to accompany ART distribution scale-up as patients transition between care centres and providers. Additionally, social support groups and counselling may help patients overcome sociocultural barriers and dispel the self-perceived stigma that scares many away from HIV therapy [8,9]. Patients will also require support and assistance to address transportation, financial constraints, and family responsibilities – structural factors that have been implicated in non-retention [8–10].

Identifying and treating prolonged viremia among PLWH failing ART can further limit population-level HIV transmission. Our results suggest that rapid detection of virological failure (e.g. through bi-annual viral load monitoring) in addition to earlier treatment could avert over 50,000 more HIV infections compared to implementing early ART initiation alone. In India, where viral load testing is not routinely available, immunological criteria are often used as a surrogate for clinical evaluation of PLWH [51]. However, CD4 cell counts have been shown to be a poor marker of virological failure and can jeopardize future therapeutic options by leading to unnecessary or untimely switches to second-line therapies [51–53]. Our model suggests that, over time, an increasing fraction of PLWH will require post-first-line therapies with earlier HIV treatment. Increased availability of viral load testing will help monitor adherence and drug resistance and guide clinical decisions on when to switch individuals to second-line therapies [51–54].

Early ART initiation is both cost-effective and affordable to the Indian healthcare system. HIV care costs are comparable between our model and current Indian expenditures. India's National AIDS Control Organization (NACO) has proposed nearly $3 billion over five years for the next phase of its HIV control programme, NACP-IV [55]. Assuming current practices, our model predicts that the Indian healthcare system will incur undiscounted costs of $2.9 billion over the next five years and $13.0 billion over 20 years. Despite increased proportions of PLWH requiring post-first-line regimens, implementation of early ART initiation will only require additional expenditures of $517 million over 20 years, representing a nominal 4.0% increase in overall Indian HIV spending.

Our study adds to a small but growing body of literature on the health benefits and cost-effectiveness of early ART initiation in India and other countries. A recent systematic review of 12 mathematical models suggested that earlier treatment was cost-effective over 20 years and should be a high-priority health intervention in low-and-middle income countries, including India [14]. However, models in this review only considered the impact of earlier treatment for targeted subpopulations within India and are thus limited in their scope. Prior models of HIV in India also did not explicitly account for the entire continuum of HIV care, such as post-linkage dropout from care that can lead to ART resistance and switches to costlier second-line therapies. Our model expands on this prior work by formally incorporating the modulatory effect of each step along the full spectrum of HIV care on policies for early ART initiation, while additionally considering the entire adult Indian population in addition to high-risk subpopulations. Furthermore, while previous studies have shown how improved screening and linkage can modify the impact of earlier treatment, our model is unique in providing the contribution of improved strengthening at each step of the HIV care continuum [14,15]. We show that improvements in care-retention are crucial to achieving population-level impact of HIV treatment.

Our model has several limitations. Costs related to potential interventions for health system strengthening were not explored, as such cost inputs are largely unknown. While we did not explicitly model specific resistance mutations, our model is among the first to specifically incorporate virological failure for standardized ART regimens and need for post-first-line therapies with earlier HIV treatment. As with all models, our epidemic-economic model simplifies complex behavioural networks and dynamics. However, we consider HIV transmission and AIDS mortality over time using a dynamic modelling framework that better accounts for transmission dynamics compared to more static decision-analytic frameworks. Furthermore, our model accurately reflects the current Indian HIV epidemic and our findings are robust over wide variation of parameters in sensitivity analysis.

In summary, early ART initiation in India provides modest benefits in averting new HIV infections and AIDS-related deaths, is highly cost-effective, and is within the financial scope of the Indian healthcare system. However, we quantitatively show that many benefits of earlier treatment are lost due to attrition throughout the HIV care continuum. Improvements in retention in care are especially required to realize the full effect of early ART initiation.

## Conclusions

Early ART initiation in India is highly cost-effective even in the context of attrition throughout the continuum of HIV care and should remain a high-priority health intervention for the Indian healthcare system. However, excess costs, HIV transmissions and AIDS deaths are projected to occur despite early ART initiation without improvements in every step of the HIV care continuum, particularly long-term retention in care. Improving retention in care should be a high-priority health intervention in India to realize the benefits of early treatment strategies.

## Supplementary Material

Economic and epidemiological impact of early antiretroviral therapy initiation in India
