# Supplementary material for: Economic and epidemiological impact of early antiretroviral therapy initiation in India
Source: J Int AIDS Soc. 2015 Oct 1;18(1):20217. doi: 10.7448/IAS.18.1.20217 (PMC4592848; doi:10.7448/IAS.18.1.20217)
Supplement: Economic and epidemiological impact of early antiretroviral therapy initiation in India [file JIAS-18-20217-s001.pdf]

Supplement

Economic and epidemiological impact of early antiretroviral therapy initiation in India

Manoj V. MADDALI[1], David W. DOWDY[2], Amita GUPTA[1,3], Maunank SHAH[1]

1. Division of Infectious Diseases, Department of Medicine, School of Medicine, Johns Hopkins University, Baltimore, MD
2. Department of Epidemiology, Bloomberg School of Public Health, Johns Hopkins University, Baltimore, MD
3. Department of International Health, Bloomberg School of Public Health, Johns Hopkins University, Baltimore, MD

We constructed an economic-epidemic model of the HIV care continuum in India. In our deterministic model, the Indian population aged 15-64 was divided by gender (male or female) and further subdivided based on HIV risk profile: heterosexuals, men who have sex with men, people who inject drugs, high-risk males, and female sex workers. High-risk males encompassed those with higher numbers of sexual partners compared to the general population, including those with higher frequency of contact with female sex workers (e.g. long-distance truck drivers or single male migrants). Population sizes for the adult Indian population and high-risk subpopulations were based on published literature from census and NACO estimates [1,2]. The population remained in the model between ages 15 and 65 to represent a 50-year duration of sexual activity. We incorporated population growth at 1.25% per year based on current census data [1]. The population was assumed to be HIV-negative at entry into the model.

HIV disease transmission

HIV transmission was modeled through heterosexual contact, male-to-male sexual contact, and needle sharing. We did not model explicit networks of sexual behavior or needle sharing, but instead transmission based on a population's average number of partnerships and average probability of transmission per partnership. For each risk-group, we began with transmission parameters based on literature estimates, but calibrated them to current observed epidemiology of HIV (i.e. HIV incidence and prevalence) in India. In addition to sexual contact, people who injected drugs could become infected with HIV through needle sharing, which was modeled based on average number of shared needles per year and probability of HIV transmission per shared needle. Transmission probabilities per sexual partnership were dependent on gender and sexual behavior (e.g. male-to-female, female-to-male, or male-to-male), stage of HIV infection (e.g. increased transmission probability per sexual partnership during acute HIV infection), ART usage (e.g. active ART usage reduced probability of transmission), and awareness of HIV serostatus (e.g. PLWH aware of HIV serostatus were less likely to engage in high-risk sexual behaviors such as unprotected sexual intercourse). We did not model female-to-female HIV transmission, due to the low likelihood of transmission per sexual partnership [3]. We further incorporated reductions in likelihood of HIV transmission per partnership based on estimates of Indian male circumcision status and condom usage within risk groups [4–7].

We allowed sexual partnerships between populations from different risk groups. Based on literature estimates, we assumed that 4% of the low-risk, heterosexual male population visited female sex workers annually [8]. Furthermore, we assumed that 50% of high-risk males were frequent clients to female sex workers with the remaining 50% serving as casual clients. Finally we incorporated low levels of mixing between MSM and heterosexual female populations and between PWID and heterosexual populations; these partnership percentages were varied widely in sensitivity analyses. To allow a balanced number of partnerships among heterosexual males and females, the average number of partnerships per year for females was calculated based on the population size and number of female partners sought by males within each risk group.

We defined the transmission rate parameter β as the average number of annual partnerships per person $n_{i,a,x,y}$ between individuals in subpopulations of gender and risk group i and their partner subpopulation a, multiplied by the probability of transmission per partnership $p_{i,a,x,y}$, where i is the gender and risk group of the index individual, a is the gender and risk group of sexual partner, x is the place in the HIV care continuum of the infectious partner (e.g. unaware or virologically suppressed), and y is the stage of HIV infection (e.g. acute HIV) of the infectious partner. The probability of transmission per partnership was modified by the probability of condom usage $\kappa_{i,a}$ with transmission reduction α as well as male circumcision status ν_i with transmission reduction $\omega_{i,a}$ for male acquitision from an infected female partner. The calculation for transmission rate β is shown in Equation 1.1.

$$\beta_{i,a,x,y} = n_{i,a,x,y}(p_{i,a,x,y}\kappa_{i,a}\alpha + (1-\kappa_{i,a}))\left[p_{i,a,x,y}v_{i,a}\omega_{i,a} + p_{i,a,x,y}(1-v_{i,a})\right] \qquad (1.1)$$

HIV care continuum in "idealistic" and "realistic" scenarios

We modeled the Indian HIV epidemic within the context of an "idealistic", optimized continuum of HIV care as well as a current care continuum that incorporates "realistic" gaps in care.

Transition of PLWH through discrete stages of the HIV care continuum within our model is described as follows: Upon acquiring HIV, PLWH become aware of their serostatus through active (e.g. symptomatic) and passive screening/testing. In the "realistic" care continuum scenario, we incorporated national rates of screening stratified by risk group from published literature; in the "idealistic" scenario, we assumed annual

screening for high-risk groups (Supplement Table 1) [2,8]. For both scenarios, we assumed NACO guidelines for HIV testing utilizing fourth generation Ag/AB HIV tests.

Once aware, PLWH either linked to care (defined as HIV clinic visit within 3 months), or remained out of care (default). In the "idealized" scenario, we assumed 95% linkage to care for all PLWH. In both scenarios, individuals linked to care were offered ART based on Indian guidelines. For the period 2007-2012, we implemented prior ART initiation at CD4 cell count ≤ 200 cells/mm^3. Beyond 2012, we assumed ART initiation in the base-case scenario (i.e. standard of care therapy) at CD4 cell count ≤ 350 cells/mm^3; we defined an early ART initiation intervention as beginning therapy at CD4 cell count ≥ 350 cells/mm^3; we assumed early ART initiation would predominantly occur at CD4 ≤ 500 cells/mm^3 as per WHO recommendations, but the model allowed for some individuals in care to initiate at higher CD4 counts. This rate of early ART initiation was varied in sensitivity analysis (Supplement Table 1) [9,10].

After initiating therapy, PLWH either experienced virological suppression and immunologic recovery or experienced virological failure. In the "realistic" care continuum, rates were based on literature estimates [11–14]. In the "idealized" scenario, we assumed better ART adherence and incorporated lower rates of virological failure. Our model incorporates first-line and second-line regimens for ART based on current Indian guidelines [9]. In the "realistic" care continuum scenario, PLWH were switched to an alternative regimen due to adverse clinical effects or virological failure at rates based on literature estimates; these rates were further stratified by time period (pre-2007, 2007-2012, and post-2012) [15,16]. In the "idealistic" care continuum, we assumed improved rates of failure identification and treatment modification (Supplement Table 1).

We considered PLWH in the linked, treated, suppressed, or treatment failure compartments to be 'in care.' PLWH could leave any stage of care (i.e. default) at a rate (l_i) based on literature estimates of retention in care [17,18]. Rates of disengagement were varied such that high-risk groups, including PWID, MSM, and FSW, were more likely to drop out of care compared to the low-risk heterosexual population. We assumed that PLWH who defaulted from care did not receive ART or other prophylactic treatments. In both "realistic" and "idealistic" care continuum scenarios, PLWH who were lost to care were eligible for reentry

into care, with the provision that 25% of those who were previously on first-line therapy had developed resistance to first-line ART and were initiated onto second-line ART upon reengagement in care [19]. We assumed that there always existed a post-first-line regimen that was available to PLWH who developed resistance to first-line regimens. We also incorporated slower treatment initiation of post-second-line regimens. Care-retention parameters in the "realistic" care continuum scenario were calibrated to reflect published estimates of the proportion of Indian PLWH retained in care after HIV diagnosis [20]. In the "idealized" continuum, we assumed minimal rates of disengagement from care and reentry into care within one year of disengagement.

HIV disease progression

In addition to an interaction with the healthcare system, upon infection, PLWH progressed through stages of HIV that were stratified by decline in CD4 count. We incorporated rates of non-opportunistic infection (non-OI) related HIV death for PLWH not on ART with increased mortality rates after AIDS diagnosis (CD4 $\leq$ 200 cells/mm^3) [21–23]. We incorporated reduced risk of HIV/AIDS related death (OI and non-OI related death) with virological suppression from ART usage [24,25]. We further incorporated OI prophylaxis for PLWH with CD4 $\leq$ 200 cells/mm^3.

HIV Dynamic Compartmental Model

Our HIV economic-epidemic model represents a dynamic system in which PLWH transition concurrently through stages of HIV disease and place in the HIV continuum of care (Supplement Figure 1). All model analysis was conducted in R version 3.1.2 (R Foundation for Statistical Computing) and MATLAB R2014a (MathWorks).

We constructed a system of linear differential equations to represent the rate of change and flow between compartments. The equations in our model were solved using generic Runge-Kutta methods in the R package deSolve with a time step of 0.1 year. We provide model equations below (for simplicity, we do not show equations for transmission due to needle sharing among PWID). S (indexed by q [awareness of serostatus] and y [stage of HIV disease]) represents the susceptible population, H (indexed by x [engagement in HIV care continuum] and y [stage of HIV disease]) represents the population infected with

HIV (except those who have disengaged from care), D (indexed by c [history of ART usage] and y [stage of HIV disease]) represents PLWH who have disengaged from care, and N represents the total population (Supplement Figure 1).

Equations 2.1 and 2.2 describe the flow dynamics of the susceptible population. $S_{q=1,i}$ represents those that are susceptible and unaware of their HIV serostatus, whereas $S_{q=2,i}$ represents the population of susceptible individuals that have had an HIV test. Persons transition from unaware to aware at screening rate σ_i that is specific to risk group and gender (i). Populations can acquire HIV from age 15 – 65, exiting the model at a sexual maturation rate of m_i. The susceptible populations also undergo a background rate of death and can exit the model at rate d_i. The susceptible population becomes infected based on transmission rate $\beta_{i,a,x,y}$ and prevalence of HIV infection among partners $\frac{H_{a,x,y}}{N_a}$ (where a is the risk group and gender of the infected partner, x is the place in the HIV care continuum, and y is the stage of HIV disease) and transitions to the HIV infection compartment ($H_{i,x=1,y=1}$).

Equations 3.1 – 3.10 describe the flow of PLWH through the HIV care continuum and Equations 4.1 – 4.3 describe PLWH who disengage from care. Equations 5.1 – 5.4 describe the progression of HIV infection or rate of death for PLWH that are not virologically suppressed ($H_{x=1–7}$ and $D_{c=1–3}$), at rates δ_y based on stage of HIV disease. Equations 5.5 – 5.7 describe rate of immunologic recovery for PLWH who are virologically suppressed.

Like all model compartments, HIV infected populations undergo background death and model exit at a rate of d_i but experience additional HIV or AIDS-related death at rate d_y. $H_{i,x=1,y=1}$ represents the population with acute HIV infection ($H_{y=1}$) who are unaware ($H_{x=1}$) of their HIV serostatus. At any stage of HIV disease ($H_{y=1–4}$), PLWH may become aware of their HIV status (transition from $H_{x=1}$ to $H_{x=2}$) either through passive screening at rates stratified by risk group and gender ($\sigma_{1,i}$) or through symptomatic presentation to the healthcare system ($\sigma_{2,i}$), as described in Equations 3.1 and 3.2.

Upon diagnosis ($H_{x=2}$), PLWH transition into linked to care ($H_{i,x=3,y}$) or lost to care ($D_{i,c=1,y}$) compartments at rate π; the proportion of PLWH that link to care (f_i) was varied by gender and risk group (i) (Equations 3.4

and 4.1). We assumed PLWH that were linked to care ($H_{x=3}$) were offered and initiated on ART per current guidelines or early ART initiation policies at rate τ_y (Equations 3.5 and 3.6). PLWH on ART ($H_{x=4–5}$) achieved virological suppression at rate ε and transitioned from $H_{x=4}$ to $H_{x=8}$ or from $H_{x=5}$ to $H_{x=9}$ (Equations 3.9 and 3.10). Populations who were virologically suppressed could experience immunological recovery at rate γ (Equations 5.5 – 5.7). PLWH on ART could also fail therapy (i.e. not achieve virological suppression) at rates μ and ϕ (failure of 1st and 2nd line ART, respectively) and could then experience treatment modification to subsequent therapies at rates ζ and χ (1st and 2nd line ART, respectively), shown in Equations 3.7 and 3.8. PLWH on 1st line ART could also experience treatment modification based on clinical symptoms at rate λ (Equation 3.5).

At any stage of interaction with the HIV care continuum, PLWH could become disengaged from care at rate l_i (varied by risk group and gender i), described by Equations 4.1 – 4.3. Disengaged populations ($D_{i,c=1–3,y}$) could reengage into care at various points at rate r, taking into account their previous exposure to ART regimens. For example, PLWH who became disengaged before ART initiation ($D_{i,c=1,y}$) reentered into care with other treatment naïve populations (Equations 3.4 and 4.1), and PLWH who previously experienced 2nd line ART regimens ($D_{i,c=3,y}$) would be reinitiated on 2nd line regimens upon reentry into care (Equations 3.6 and 4.3). A proportion of PLWH on 1st line ART who disengaged from care ($D_{i,c=2,y}$) could develop resistance to 1st line regimens with probability ψ and require initiation of subsequent regimens (Equations 3.5, 3.6, and 4.2).

HIV transmission when susceptible (Equations 2.1 – 2.2)

Unaware

$$dS_{q=1,i}/dt = bNz_i - \sum_{x=1}^{9}\sum_{y=1}^{4}\sum_{a=1}^{8}\beta_{i,a,x,y}S_{1,i}\frac{H_{a,x,y}}{N_a}\ldots \quad (2.1)$$
$$\ldots-(\sigma_{1,i}+m_i+d_i)S_{1,i}$$

Aware

$$dS_{q=2,i}/dt = -\sum_{x=1}^{9}\sum_{y=1}^{4}\sum_{a=1}^{8}\beta_{i,a,x,y}S_{2,i}\frac{H_{a,x,y}}{N_a}\ldots \quad (2.2)$$
$$\ldots+\sigma_{1,i}S_{1,i}-(m_i+d_i)S_{2,i}$$

HIV care continuum engagement for all PLWH except those disengaged from care (Equations 3.1 – 3.10)

Acute HIV, Unware

$$dH_{i,x=1,y=1}\Big/dt = \sum_{x=1}^{9}\sum_{y=1}^{4}\sum_{a=1}^{8}\beta_{i,a,x,y}(S_{1,i}+S_{2,i})\frac{H_{a,x,y}}{N_a}\ldots \tag{3.1}$$
$$\ldots-(\sigma_{1,i}+\sigma_{2,i}+m_i+d_i+d_y)H_{i,1,1}$$

Unware

$$dH_{i,x=1,y=2-4}\Big/dt = -(\sigma_{1,i}+\sigma_{2,i}+m_i+d_i+d_y)H_{i,1,y} \tag{3.2}$$

Aware

$$dH_{i,x=2,y}\Big/dt = (\sigma_{1,i}+\sigma_{2,i})H_{i,1,y}-(\pi_i+m_i+d_i+d_y)H_{i,2,y} \tag{3.3}$$

Linked to care

$$dH_{i,x=3,y}\Big/dt = (\pi_i f_i)H_{i,2,y}+rD_{i,1,y}-(\tau_y+l_i+m_i+d_i+d_y)H_{i,3,y} \tag{3.4}$$

On 1st line ART

$$dH_{i,x=4,y}\Big/dt = \tau H_{i,3,y}+r(1-\psi)D_{i,2,y}-(\lambda+l_i+\varepsilon+\mu+m_i+d_i+d_y) \tag{3.5}$$

On 2nd line ART

$$dH_{i,x=5,y}\Big/dt = \lambda H_{i,4,y}+\zeta H_{i,6,y}+\chi H_{i,7,y}+r(\psi D_{i,2,y}+D_{i,3,y})\ldots \tag{3.6}$$
$$\ldots-(l_i+\varepsilon+\phi+m_i+d_i+d_y)H_{i,5,y}$$

Failing 1st line ART

$$dH_{i,x=6,y}\Big/dt = \mu H_{i,4,y}-(\zeta+l_i+m_i+d_i+d_y)H_{i,6,y} \tag{3.7}$$

Failing 2nd line ART

$$dH_{i,x=7,y}\Big/dt = \phi H_{i,5,y}-(\chi+l_i+m_i+d_i+d_y)H_{i,7,y} \tag{3.8}$$

Suppressed on 1st line ART

$$dH_{i,x=8,y}\Big/dt = \varepsilon H_{i,x=4,y}-(\mu+l_i+m_i+d_i+d_y)H_{i,8,y} \tag{3.9}$$

Suppressed on 2nd line ART

$$dH_{i,x=9,y}\Big/dt = \varepsilon H_{i,x=5,y}-(\phi+l_i+m_i+d_i+d_y)H_{i,9,y} \tag{3.10}$$

HIV care continuum engagement for PLWH disengaged from care (Equations 4.1 – 4.3)

Disengaged before ART initiation

$$dD_{i,c=1,y}\Big/dt = \pi_i(1-f_i)H_{i,2,y}+l_iH_{i,3,y}-(r+m_i+d_i+d_y)D_{i,1,y} \tag{4.1}$$

Disengaged after 1st line ART initiation

$$dD_{i,c=2,y}\Big/dt = l_i(H_{i,4,y}+H_{i,6,y}+H_{i,8,y})-(r+m_i+d_i+d_y)D_{i,2,y} \tag{4.2}$$

Disengaged after 2nd line ART initiation

$$dD_{i,c=3,y}\Big/dt = l_i(H_{i,5,y}+H_{i,7,y}+H_{i,9,y})-(r+m_i+d_i+d_y)D_{i,3,y} \tag{4.3}$$

HIV progression for PLWH (equations 5.1 – 5.7)

(For simplicity, we do not show HIV disease progression for those disengaged from care)

Acute HIV

$$dH_{i,x=1:4,y=1}\Big/dt = -\delta_1(H_{i,x,1}) \tag{5.1}$$

Early HIV, Unsuppressed
$$\left. dH_{i,x=1:7,y=2} \middle/ dt \right. = \delta_1(H_{i,x,1}) - \delta_2(H_{i,x,2}) \tag{5.2}$$

Late HIV, Unsuppressed
$$\left. dH_{i,x=1:7,y=3} \middle/ dt \right. = \delta_2(H_{i,x,2}) - \delta_3(H_{i,x,3}) \tag{5.3}$$

AIDS, Unsuppressed
$$\left. dH_{i,x=1:7,y=4} \middle/ dt \right. = \delta_3(H_{i,x,3}) - \delta_4(H_{i,c,4}) \tag{5.4}$$

Early HIV, Suppressed
$$\left. dH_{i,x=8:9,y=2} \middle/ dt \right. = \gamma_1 H_{i,x,3} \tag{5.5}$$

Late HIV, Suppressed
$$\left. dH_{i,x=8:9,y=3} \middle/ dt \right. = \gamma_2 H_{i,x,4} - \gamma_1 H_{i,x,3} \tag{5.6}$$

AIDS, Suppressed
$$\left. dH_{i,x=8:9,y=4} \middle/ dt \right. = -\gamma_2 H_{i,x,4} \tag{5.7}$$

Model Initiation and Calibration

To determine the initial populations in each model compartment, we first brought the model to equilibrium in 2007 and then allowed population growth at an annual rate of 1.25% per year [1]. We calibrated model outputs until 2011, the last year in which data was available. During model initiation and calibration, all parameters except transmission probabilities (e.g. number of partners and transmission probability per partnership) and care-retention parameters were held fixed (Table 1). Virological benefits of ART were also adjusted to reflect the improved efficacy of ART regimens after 2011. Beyond 2014, standard of care therapy was incorporated as ART initiation at CD4 ≤ 350 cells/mm^3 [9].

To determine transmission probabilities, we calibrated partner rates and transmission probabilities per partnership to generate model outputs that closely approximate epidemiological data on HIV prevalence and incidence from 2007 to 2011 through an iterative process [26]. We further calibrated parameters (π, l, and R) related to retention in care to reflect current estimates of the HIV continuum of care in India [17,18,20]. Model outputs of PLWH in India and engagement in the Indian HIV care continuum in 2014 are shown in Supplement Figure 1 and 2.

Costs and QALYs

Total health system costs for the adult Indian population were calculated based on person-time spent in each compartment as well as the number of transitions between compartments; individual costs were varied

by HIV status and place in the HIV care continuum. Future costs were discounted by 3% per year, though we also provide undiscounted costs. We included costs for Voluntary Counseling and Testing (VCT) at rates based on published estimates of national HIV screening (Table 1) [8]. We also incorporated HIV testing for PLWH presenting with symptoms to healthcare facilities. For PLWH who linked to care, we accounted for costs of baseline CD4 count, HIV viral load prior to ART initiation, and costs for HIV clinic visit; we accounted for similar costs for PLWH returning to care after disengagement (Supplement Table 2) [2]. We assumed two HIV clinic visits occur per year with HIV viral load monitoring occurring annually. We based costs for antiretroviral therapy on current Indian guidelines, with a first-line therapy of tenofovir, lamivudine, and efavirenz (TDF/3TC/EFV) and second-line therapy of zidovudine, lamivudine, and lopinavir/ritonavir (AZT/3TC/LPV/r) [9]. We further included costs for care of PLWH not on ART to account for hospitalizations, ED visits, and other increased healthcare utilization (Supplement Table 2). We estimated unit costs for VCT, CD4 count, HIV viral load monitoring, first and second-line antiretroviral therapy, clinic visits and hospitalizations, and other healthcare expenditures based on published literature [27–30].

Total Quality-adjusted life-years (QALYs) accumulated by the adult Indian population were generated based on person-time spent in the model with and without HIV/AIDS and with and without ART (e.g. persons not infected with HIV accumulated 1 QALY per year spent in the model until exit or death). Future QALYs were discounted by 3% per year. Utility weights are provided in Table 1.

Additional Sensitivity Analysis

We conducted additional sensitivity analyses to determine key determinants of the economic and economic impact of early ART initiation in India within the context of a "realistic" continuum of care. Incremental cost of an early ART initiation policy was strongly influenced by cost of 1st and 2nd line ART regimens and by rate of failure of ART, as well as retention in care (Supplement Figure 4). Rate of symptomatic HIV screening and yearly disengagement from care were key determinants for the number of new HIV infections averted as a result of early ART initiation (Supplement Figure 5). The number of AIDS deaths averted through early ART initiation was impacted by the rate of treatment modification after failure and

symptomatic HIV screening (Supplement Figure 6). Incremental costs, infections averted, and deaths averted were all influenced by the rate of ART initiation at CD4 ≥350 cells/mm^3. Additionally, we generated a cost-effectiveness acceptability curve from the probabilistic sensitivity analysis that suggests that early ART initiation in India is cost-effective even with a broad range of willingness-to-pay thresholds (Supplementary Figure 7).

Supplement Figures

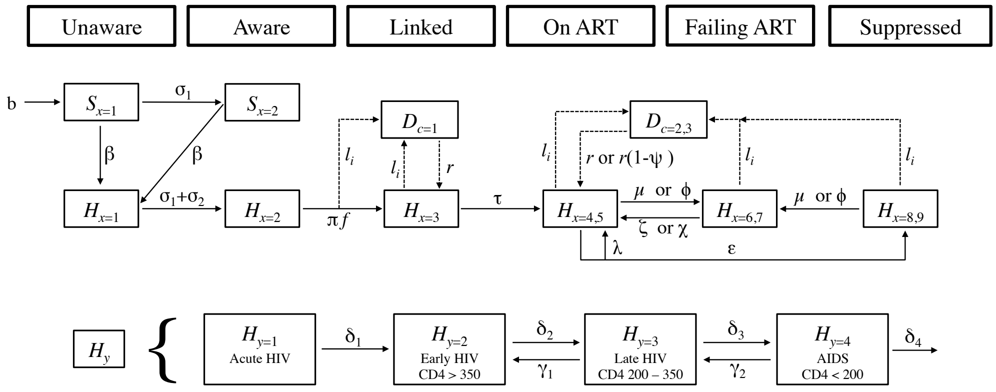

Supplement Figure 1: Model schematic of compartmental model with care continuum and HIV disease dynamics

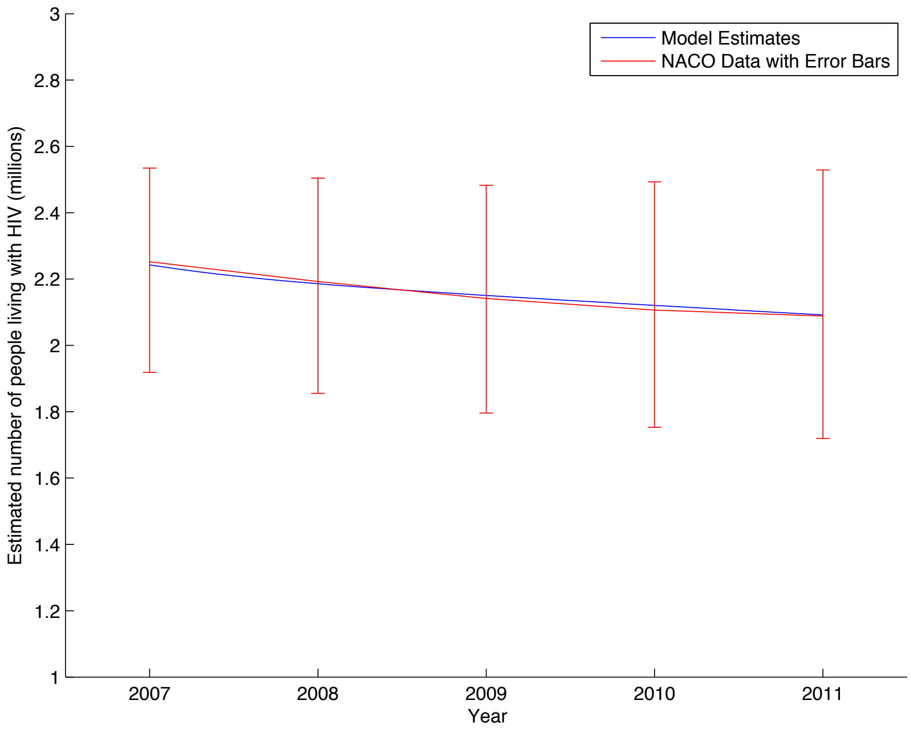

Supplement Figure 2: Model calibration at baseline with estimates of number of people living with HIV (PLWH)

Figure legend: Blue line represents model output of number of PLWH; red line and error bars indicate NACO estimates of PLWH [26].

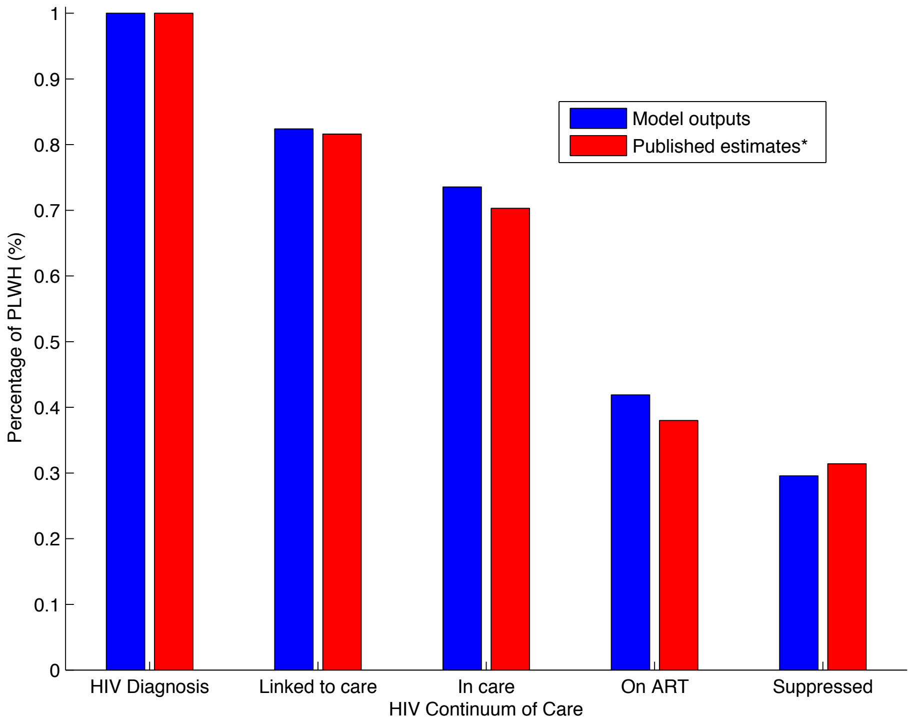

Supplement Figure 3: Baseline model estimates of continuum in care (pre-2014).

Figure legend: Average baseline model estimates of continuum in care. Blue bars represent model outputs, while red bars represent published estimates [20].

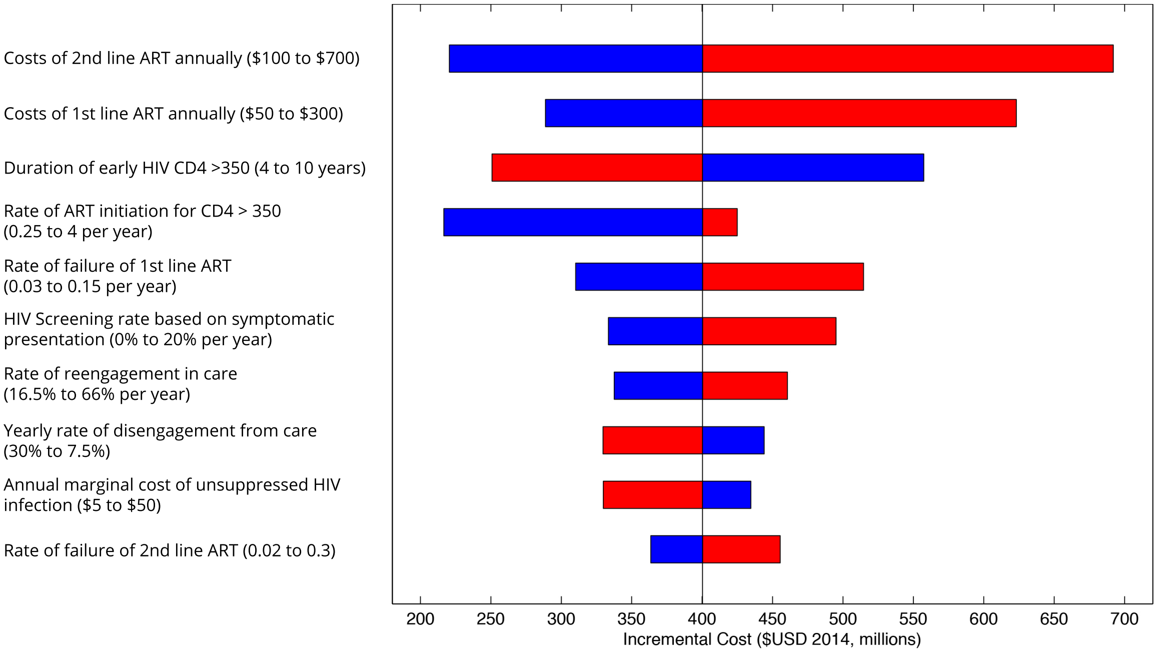

Supplement Figure 4: Sensitivity analysis for incremental cost-effectiveness ratio incurred by implementing early ART initiation within the context of a "realistic" continuum of care, compared with current practices of ART initiation (CD4 ≤350 cells/mm^3).

Figure legend: Sensitivity analysis of key parameters impacting incremental healthcare costs incurred by implementing early ART initiation within the context of a "realistic" continuum of care, compared with current practices for ART initiation (CD4 ≤ 350 cells/mm^3). Solid vertical line represents base incremental healthcare cost ($400 million). Blue bars indicate low values of parameter range; red bars indicate high values of parameter range.

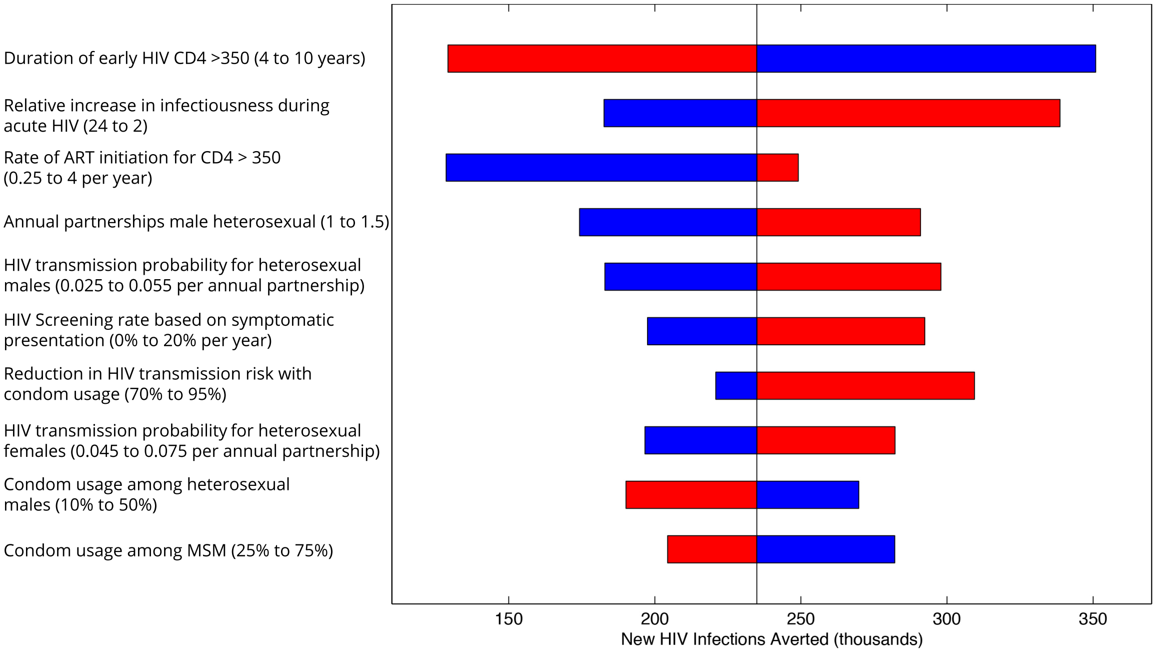

Supplement Figure 5: Sensitivity analysis for new HIV infections averted by implementing early ART initiation within the context of a "realistic" continuum of care, compared with current practices of ART initiation (CD4 ≤350 cells/mm^3).

Figure legend: Sensitivity analysis of key parameters impacting new HIV infections averted by implementing early ART initiation within the context of a "realistic" continuum of care, compared with current practices for ART initiation (CD4 ≤ 350 cells/mm^3). Solid vertical line represents base new infections averted (235,000). Blue bars indicate low values of parameter range; red bars indicate high values of parameter range.

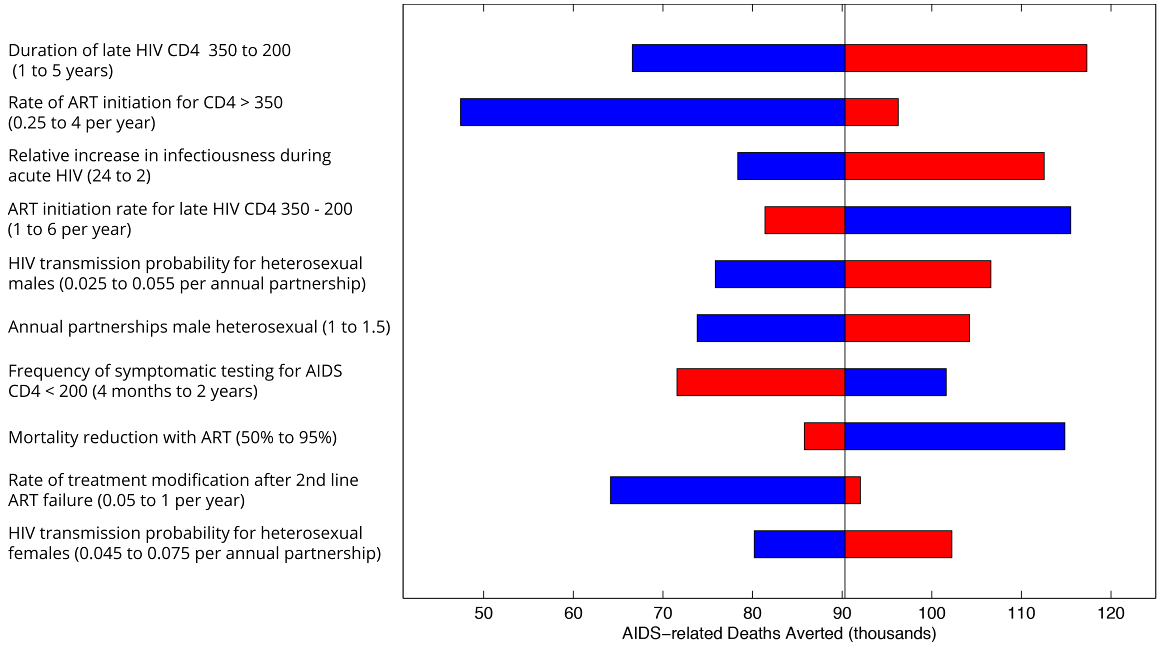

Supplement Figure 6: Sensitivity analysis for AIDS-related deaths averted by implementing early ART initiation within the context of a "realistic" continuum of care, compared with current practices of ART initiation (CD4 ≤350 cells/mm^3).

Figure legend: Sensitivity analysis of key parameters impacting AIDS deaths averted by implementing early ART initiation within the context of a "realistic" continuum of care, compared with current practices for ART initiation (CD4 ≤ 350 cells/mm^3). Solid vertical line represents base AIDS-related deaths averted (90,000). Blue bars indicate low values of parameter range; red bars indicate high values of parameter range.

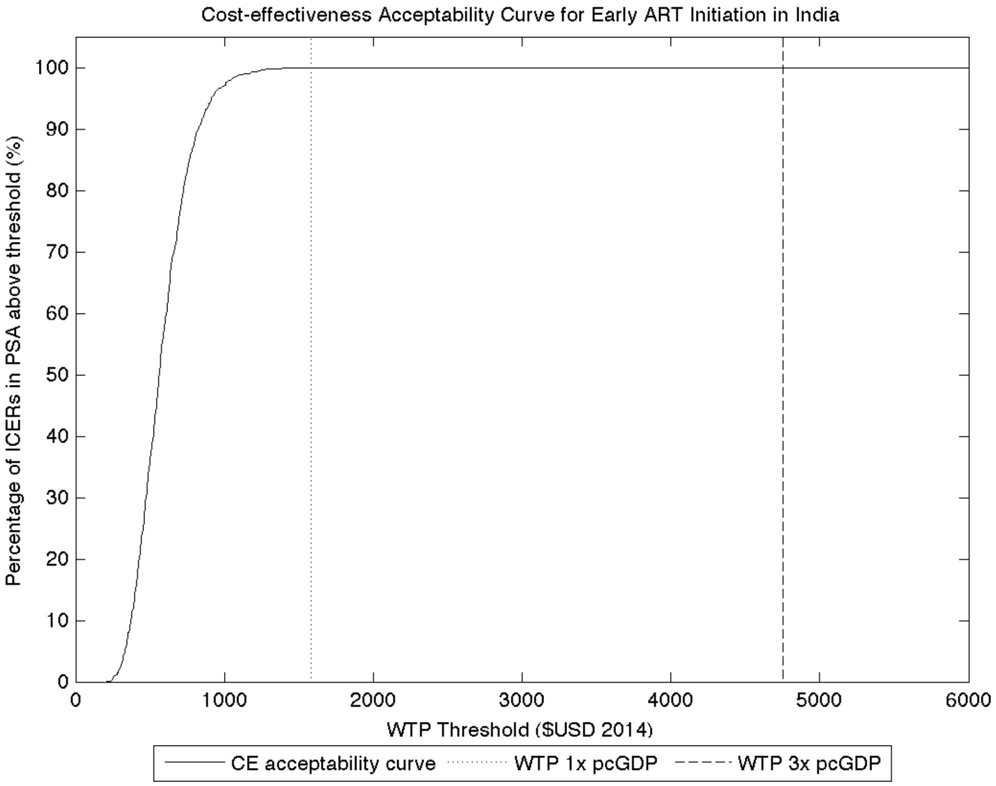

Supplement Figure 7: Cost-effectiveness acceptability curve for early ART initiation in India compared to current initiation at CD4 ≥350 cells/mm^3

Figure legend: Cost-effectiveness acceptability curve calculated from probabilistic sensitivity analysis comparing early ART initiation in India with current practices for ART initiation (CD4 ≤350 cells/mm^3) in a "realistic" continuum of care. Dotted line represents willingness-to-pay threshold of "very cost-effective" or less than 1x per capita GDP, and dashed line represents willingness-to-pay threshold of "cost-effective" or less than 3x per capita GDP (2014 Indian GDP per capita is $1,584).

Supplement Table 1: Key model parameter descriptions and values within the context of a current, "realistic" continuum of care*.

Variable	Description
i	Gender (1 male, 2 female) and risk-group (heterosexual, MSM, PWID, high-risk male, FSW) of index compartment [see Supplement Table 1].
a	Gender (1 male, 2 female) and risk-group (heterosexual, MSM, PWID, high-risk male, FSW) of partner compartment [see Supplement Table 1].
x	Place in HIV care continuum for PLWH who are not disengaged from care (1 Unaware, 2 Aware, 3 Linked to care, 4 On 1st line treatment, 5 On 2nd line treatment, 6 Failing 1st line treatment, 7 Failing 2nd line treatment, 8 Suppressed with 1st line treatment, 9 Suppressed with 2nd line treatment, 10 Aware of serostatus but disengaged from care) (Supplement Figure 1).
y	Stage of HIV infection (1 Acute HIV, 2 CD4 ≥ 350, 3 CD4 200 – 350, 4 CD4 ≤ 200) (Supplement Figure 1).
q	Awareness status of HIV-susceptible individuals (1 Unaware, 2 Aware)
c	History of ART exposure for PLWH disengaged from care (1 Treatment naïve, 2 Experienced 1st line ART, 3 Experienced 2nd line ART)
$N(t)$	Number of individuals in the population at time t. We assumed recent census population estimates for the following groups: Total adult population 15-64 years (812,000,000) [1]; People who inject drugs [PWID] – Men (159,300) [2]; People who inject drugs [PWID] – Female (17,700) [2]; Men who have sex with men [MSM] (412,000) [2]; Female sex workers [FSW] (868,000) [2]; High-risk males encompassed 1% of the entire population [1]
$S_q(t)$	Number of susceptible individuals in compartment q at time t.
$H_x(t)$	Number of PLWH (except those disengaged from care) in compartment x at time t.
$D_c(t)$	Number of PLWH disengaged from care in compartment c at time t.
$n_{i,a,x,y}$	Average number of sexual partners between persons of gender and risk group i with partners of gender and risk group a at HIV care continuum engagement x and HIV disease stage y.
$p_{i,a,x,y}$	Probability of sexual transmission of HIV between susceptible persons of gender and risk group i and infectious partner of gender and risk group a at HIV care continuum engagement x and HIV disease stage y.
$\varkappa_{i,a}$	Probability of condom usage among partnerships between risk and gender groups i and a (heterosexual 25% [range 10% – 50%]; MSM 50% [range 25% – 75%]; high-risk males 15% [range 5% – 30%]; FSW 75% [range 50% – 95%]) [8,31], assumption.
A	Efficacy of condom usage in reducing HIV transmission (90% [range 70% – 95%]) [4].
$\nu_{i,a}$	Percent male circumcision among susceptible males and/or infectious male partners (10% of males [range 1% – 50%]) [5].
$\omega_{i,a}$	Efficacy of male circumcision in reducing HIV transmission between individuals of gender i and partner a when either the susceptible or infectious partner is HIV positive; we assumed no protection for women with an infected circumcised male partner (60% reduction in transmission for male acquisition from female partner [range 10% – 60%], 10% reduction in acquisition with MSM partner [range 10% – 40%] [6,7].
b	Entry rate.
z_i	Proportion of total population made up of persons of risk group and gender i.
m_i	Rate of sexual maturation (e.g. 1/duration, 50 years for all Indian adults).
d_i	Rate of background death.
d_y	HIV mortality at HIV disease stage y.
σ_i	$\sigma_{1,i}$ represents annual HIV screening rate among persons of gender and risk group i. (heterosexual male/female 3.2% [range 1% – 7.5%], MSM 17% [range 5% – 30%], PWID 20.7% [range 10% – 40%], high-risk males 3.2% [range 1% – 7.5%], FSW 31.8% [range 10% – 60%]) [2,4].<br>$\sigma_{2,i}$ represents rate of symptomatic HIV testing (5% per year CD4 > 200 [range 0% – 20%], diagnosis within 1 year for CD4 ≤ 200 [range diagnosis within 6 months – 3 years]) [2].
π_i	Rate of linkage to care (3 months [range 2 months – 1 year]).
f_i	Percent of PLWH of risk group and gender i with early linkage to care (heterosexual male 80% [range 60% – 100%], heterosexual female 80% [range 60% – 100%], MSM 70% [range 50% – 90%], PWID male 50% [range 25% – 75%], PWID female 55% [range 25% – 75%], high-risk male 70% [range 50% – 90%], FSW 80% [range 60% – 100%]) [20, assumption].
τ_y	Rate of ART initiation dependent on CD4 count. In base-case (current standard of care): 0 per year for CD4 > 350, 3 per year for CD4 ≤ 350, 6 per year for CD4 ≤ 200. In early ART initiation intervention: 2 per year for CD4 > 350, 3 per year for CD4 ≤ 350, 6 per year for CD4 ≤ 200 [9,10, calculated].
ε	Rate of virologic suppression upon ART initiation: suppression within 4 months [range suppression within 2 months – 1 year] [11,12].
μ	Rate of failure of 1st line ART: 0.07 per year (range 0.03 – 0.15 per year) [15,16, calculated].
ϕ	Rate of failure of 2nd line ART: 0.10 per year (range 0.02 – 0.30 per year) [15,16, calculated].
ψ	Percent of PLWH who develop resistance to 1st line ART upon disengagement: 25% (range 10% – 50%) [19, assumption].
ζ	Rate of detection of failure of 1st line ART and subsequent treatment modification: 0.8 per year (range 0.5 – 1.5 per year) [15,16, calculated].
χ	Rate of detection of failure of 2nd line ART and subsequent treatment modification: 0.5 per year (range 0.05 – 1.0 per year) [15,16, calculated].
λ	Rate of treatment modification based on clinical symptoms: 0.05 per year (range 0.01 – 0.1 per year) [15,16, calculated].
l_i	Rate of yearly disengagement from care (i.e. loss to follow-up) among those in HIV care, stratified by gender and risk group i: heterosexual male 0.15; heterosexual female 0.15; MSM 0.15, PWID male 0.165, PWID female 0.15, high-risk male 0.18 FSW 0.195; uncertainty range for all 0.075 – 0.3 [17,18, calculated].
r	Rate of reengagement in care among those aware of HIV status but not in care: 0.33 per year [range 0.165 – 0.66] [17,18,

	calculated].
**δ**	δ_1 represents rate of progression from acute HIV (i.e. 1/duration of acute HIV); δ_2 represents rate of progression from baseline CD4 to CD4 ≤ 350; δ_3 represents rate of progression from CD4 ≤ 350 to CD4 ≤ 200; δ_4 represents rate of progression to death among individuals with CD4 ≤ 200.
**γ**	Rate of immunological recovery on ART with viral suppression (e.g. transition from CD4 ≤ 200 to CD4 200 – 350 to CD4 > 350): 0.6 per year [13,14, calculated].

*In the "idealistic" continuum of care, we incorporated annual HIV screening (σ_i=1) for high-risk populations, 95% linkage to care for all PLWH after HIV diagnosis (f_i=95%), lower rates of ART failure due to improved adherence (μ=0.03 *and* ϕ=0.05 per year), and optimal retention in care with low rates of disengagement and reentry into care within one year of disengagement (l_i=0.025 per year and r=1.0).

Supplement Table 2: HIV care costs and additional model parameters

**Costs ($USD, 2014)**	**Value**	**Sensitivity Analysis**	**References**
Voluntary counseling and testing (VCT)	$4.74	$1 – $10	[27]
CD4 test	$6.63	$3 – $15	[28]
HIV viral load	$48.65	$20 – $100	[28]
Outpatient clinic visit	$3.17	$1 – $15	[29]
Annual first-line ART cost	$133.40	$50 – $300	[30, calculated]
Annual second-line ART cost	$328.80	$100 – $700	[30, calculated]
Cost of increased annual healthcare utilization (inpatient, ED) for PLWH CD4 > 200 (untreated HIV)	$19.75	$5 – $50	[29, calculated]
Cost of increased annual healthcare utilization (inpatient, ED) for PLWH CD4 ≤ 200 (untreated AIDS)	$159.31	$50 – $500	[29, calculated]

References

1. World Factbook. Central Intelligence Agency. 2014.
2. National AIDS Control Organization. NACO Annual Report 2013–14. India: NACO; 2014.
3. Petersen LR, Doll L, White C, Chu S. No evidence for female-to-female HIV transmission among 960,000 female blood donors. The HIV Blood Donor Study Group. *J Acquir Immune Defic Syndr* 1992; 5: 853–5.
4. Weller SC, Davis-Beaty K. Condom effectiveness in reducing heterosexual HIV transmission. *Cochrane Database of Systematic Reviews* 2002. Issue 1.
5. Bharat S, Kharwal S. Male circumcision within the context of HIV prevention in India: a qualitative study conducted among young men in Chandigarh, India. *IOSR-JHNS* 2013.
6. Sánchez J, Sal Y Rosas VG, Hughes JP, *et al*. Male circumcision and risk of HIV acquisition among MSM. *AIDS* 2011; 25: 519–23.
7. Reynolds SJ, Shepherd ME, Risbud AR, *et al*. Male circumcision and risk of HIV-1 and other sexually transmitted infections in India. *Lancet* 2004; 363: 1039–40.
8. Country Progress Report: India. UN Special Session of the General Assembly (UNGASS). March 2010.
9. National AIDS Control Organization (NACO). Antiretroviral guidelines for HIV-infected adults and adolescents. India: NACO; 2013.
10. Consolidated Guidelines on the Use of Antiretroviral Drugs for Treating and Preventing HIV Infection: Recommendations for a Public Health Approach. World Health Organization. June 2013.
11. Phillips AN, Staszewski S, Weber R, *et al*. HIV viral load response to antiretroviral therapy according to the baseline CD4 cell count and viral load. *JAMA* 2001; 286: 2560–7.
12. Currie S, Rogstad KE, Piyadigamage A, Herman S. Time taken to undetectable viral load, following the initiation of HAART. *Int J STD AIDS* 2009; 20: 265–6.
13. Gras L, Kesselring AM, Griffin JT, *et al*. CD4 cell counts of 800 cells/mm3 or greater after 7 years of highly active antiretroviral therapy are feasible in most patients starting with 350 cells/mm3 or greater. *J Acquir Immune Defic Syndr* 2007; 45: 183–92.
14. Mocroft A, Phillips AN, Ledergerber B, *et al*. Estimated average annual rate of change of CD4(+) T-cell counts in patients on combination antiretroviral therapy. *Antivir Ther (Lond)* 2010; 15: 563–70.
15. Zhou J, Li PCK, Kumarasamy N, *et al*. Deferred modification of antiretroviral regimen following documented treatment failure in Asia: results from the TREAT Asia HIV Observational Database (TAHOD). *HIV Med* 2010; 11: 31–9.
16. Boettiger DC, Kerr S, Ditangco R, *et al*. Trends in first-line antiretroviral therapy in Asia: results from the TREAT Asia HIV observational database. *PLoS ONE* 2014; 9: e106525.
17. Blutinger EJ, Solomon S, Srikrishnan AK, *et al*. Dropout from care among HIV-infected patients enrolled in care at a tertiary HIV care center in Chennai, India. *AIDS Care* 2014; 26: 1500–5.
18. Zhou J, Tanuma J, Chaiwarith R, *et al*. Loss to Followup in HIV-Infected Patients from Asia-Pacific Region: Results from TAHOD. *AIDS Res Treat* 2012; 2012: 375217–10.
19. Gupta A, Saple DG, Nadkarni G, *et al*. One-, two-, and three-class resistance among HIV-infected patients on antiretroviral therapy in private care clinics: Mumbai, India. *AIDS Res Hum Retroviruses* 2010; 26: 25–31.
20. Alvarez-Uria G, Pakam R, Midde M, Naik PK. Entry, Retention, and Virological Suppression in an HIV Cohort Study in India: Description of the Cascade of Care and Implications for Reducing HIV-Related Mortality in Low- and Middle-Income Countries. *Interdiscip Perspect Infect Dis* 2013; 2013: 384805–8.
21. Antiretroviral Therapy Cohort Collaboration. Life expectancy of individuals on combination antiretroviral therapy in high-income countries: a collaborative analysis of 14 cohort studies. *Lancet* 2008; 372: 293–9.
22. Strategies for Management of Antiretroviral Therapy (SMART) Study Group, Emery S, Neuhaus JA, *et al*. Major clinical outcomes in antiretroviral therapy (ART)-naive participants and in those not receiving ART at baseline in the SMART study. *J Infect Dis* 2008; 197: 1133–44.
23. Study Group on Death Rates at High CD4 Count in Antiretroviral Naive Patients, Lodwick RK, Sabin CA, *et al*. Death rates in HIV-positive antiretroviral-naive patients with CD4 count greater

than 350 cells per microL in Europe and North America: a pooled cohort observational study. *Lancet* 2010; 376: 340–5.

24. Survival after introduction of HAART in people with known duration of HIV-1 infection. The CASCADE Collaboration. Concerted Action on SeroConversion to AIDS and Death in Europe. *Lancet* 2000; 355: 1158–9.
25. Murphy EL, Collier AC, Kalish LA, *et al*. Highly active antiretroviral therapy decreases mortality and morbidity in patients with advanced HIV disease. *Ann Intern Med* 2001; 135: 17–26.
26. NACO Technical Report: HIV Estimates 2012. Indian National AIDS Control Organization (NACO). November 2012.
27. Dandona L, Kumar SP, Ramesh Y, *et al*. Changing cost of HIV interventions in the context of scaling-up in India. *AIDS* 2008; 22 Suppl 1: S43–9.
28. Venkatesh KK, Becker JE, Kumarasamy N, *et al*. Clinical impact and cost-effectiveness of expanded voluntary HIV testing in India. *PLoS ONE* 2013; 8: e64604.
29. Unit cost estimates for service delivery. World Health Organization-CHOICE. July 2011.
30. World Health Organization. Global Price Reporting Mechanism for HIV. December 2013.
31. Bradley J, Moses S, Blanchard JF, Rajaram S, Ramesh BM, Verma S, *et al*. Assessing reported condom use among female sex workers in southern India through examination of condom availability. *Sex Transm Infect* 2010; 86 Suppl 1:i44–8.
